# Epstein Barr Virus (EBV) Latent Membrane Protein 1 (LMP-1) Regulates Functional Markers in Intermediate and Non-Classical Monocytes

**DOI:** 10.3390/cancers16244169

**Published:** 2024-12-14

**Authors:** Agustina Moyano, Ana Colado, María Eugenia Amarillo, Elena De Matteo, María Victoria Preciado, Mercedes Borge, Paola Chabay

**Affiliations:** 1 Multidisciplinary Institute for Investigation in Pediatric Pathologies (IMIPP), CONICET-GCBA, Pathology Division, Ricardo Gutiérrez Children’s Hospital, Buenos Aires C1425EFD, Argentina; agus.moyano@hotmail.com (A.M.); maria.eugenia.amarillo41@gmail.com (M.E.A.); elenadematteo@gmail.com (E.D.M.); preciado@conicet.gov.ar (M.V.P.); 2 Laboratory of Oncological Immunology, Institute of Experimental Medicine (IMEX), CONICET-National Academy of Medicine (ANM), Buenos Aires C1425ASU, Argentina; anita_colado@hotmail.com (A.C.); mercedesborge@hotmail.com (M.B.)

**Keywords:** EBV, pediatric, monocytes, macrophages, tonsil

## Abstract

**Background**: The Epstein–Barr virus (EBV) infects more than 90 percent of the human population. In pediatric patients, the innate immune response against EBV primary infection plays a key role. Monocytes and macrophages can have distinct functions depending on the microenvironment surrounding them. At least three monocyte subpopulations can be differentiated depending on membrane protein expression: classical (C, CD14++CD16−), intermediate (I, CD14++CD16+), and non-classical (NC, CD14+CD16++). They also modulate T and B lymphocyte activation/inhibition through the expression of costimulatory molecules such as CD80, CD86, and PD-L1. Yet, little is known about monocytes’ role in EBV infection. **Methods**: Peripheral blood and tonsil biopsies of EBV primary infected (PI) patients, healthy carriers (HCs), and patients undergoing reactivation (R) were studied. **Results**: Classical monocytes prevailed in all infectious statuses. Tonsillar CD163 positively correlated with CD163 expression in NC monocytes in HCs. PD-L1+ cells in the tonsil positively correlated with PD-L1 expression in NC monocytes. LMP-1 viral latent protein presented a positive correlation with PD-L1, CD163, and CD206 expression in the NC subpopulation. **Conclusions**: Our results evidence the predominant role of I and NC monocytes’ response against EBV infection. Furthermore, the viral oncoprotein LMP-1 could be involved in the expression of regulatory proteins in I and NC monocytes.

## Simple Summary

Most studies on EBV primary infection have been performed in adolescents and young adult populations with infectious mononucleosis (IM) in developed countries. Furthermore, studies related to macrophage polarization were assessed in EBV-associated lymphomas. Little is known about monocytes as macrophage precursors in the context of primary infection. Here, our aim was to characterize peripheral blood monocytes, evaluate their behavior in EBV asymptomatic infection, and associate them with macrophage findings, to further comprehend the role of monocytes and macrophages in EBV pediatric infection. These findings may contribute to explaining, at least in part, the asymptomatic viral infection in children from an underdeveloped region, since specific subpopulations of monocytes (intermediate and non-classical) might have a predominant role in the response against EBV infection. Furthermore, the viral oncoprotein LMP-1 could influence the expression of regulatory proteins by these monocytes, generating a permissive environment for viral infection.

## 1. Introduction

The Epstein–Barr virus (EBV) was discovered in 1964 in a Burkitt lymphoma and was the first human virus associated with tumors. The EBV is a gamma herpesvirus that infects more than 90 percent of the human population. It disseminates through saliva and, given its tropism for B lymphocytes, primary infection (PI) occurs in the tonsil, where the virus has access to the target cell. After PI, the virus establishes a lifelong persistent infection as an episome in the memory B cell [1]. The EBV can establish two life cycles: the latent and the lytic cycle. When lytic genes are expressed, the replicative phase allows the virus to spread. In the latency cycle, few genes are expressed, and the virus persists silently in host cells, avoiding immune recognition [2]. Immune responses against EBV PI have different characteristics depending on the age of infection. When it comes to adults, there is a significant proliferation of the CD8+ compartment, responsible for the development of a characteristically febrile illness known as infectious mononucleosis (IM) [3]. When PI occurs in pediatric patients, there is no disruption of the lymphocyte subset, and it has been hypothesized that the innate immune system may play a key role in the response against the virus, preventing the development of symptoms [4]. Asymptomatic infection is characteristic of undeveloped countries. In fact, in Argentina, 90% of 3-year-old children have positive serology for EBV [5]. In addition, in our country, it has been demonstrated that the prevalence of different pediatric lymphomas associated with EBV (Hodgkin, Burkitt, and diffuse large B cell lymphoma) was statistically higher in children younger than 10 years old [6]. Although the mechanism by which the EBV is associated with tumor development is not completely understood, several viral proteins modulate the immune response [7]. Moreover, the lytic viral gene BCRF-1 encodes a viral homolog of the human IL-10, a potent anti-inflammatory cytokine, and BARF1, which inhibit M-CSF and restrain monocyte differentiation and macrophage function, favoring an immune escape [8,9].

Monocytes and macrophages are extremely plastic cells; they can have distinct, even opposite functions, depending on the microenvironment surrounding them. At least three monocyte subpopulations can be differentiated depending on the expression of CD14 and CD16 membrane proteins: classical (C, CD14++CD16−), intermediate (I, CD14++CD16+), and non-classical (NC, CD14+CD16++). Classical subpopulation represents 80–90% of the total circulating monocytes. They have phagocytic activity; produce ROS; and secret cytokines and chemokines such as IL-10, IL-6, IL-8, CCL2, and CCL5 in response to bacteria [10]. Intermediate monocytes have high antigen presentation activity and participate in the inflammatory response against bacteria by secreting TNF-α. Non-classical monocytes have a key role as housekeepers patrolling the endothelium, and they exert specific effector functions in response to viruses, given their ability to produce inflammatory cytokines such as TNF-α, IL-1β, and CCL3 [11]. The three subpopulations of monocytes differ in quantity and functionality, and together, they play a key role in the articulation of the innate-adaptive immune response since they have the ability to directly modulate T and B lymphocyte activation/inhibition through the expression of costimulatory molecules such as CD80, CD86, and PD-L1 [12]. In addition, monocytes that migrate into tissues can differentiate into macrophages, which acquire different phenotypes depending on the stimuli they receive from the microenvironment. Broadly, macrophages can be polarized into two major profiles, the classically activated (M1, CD68+) macrophages with proinflammatory functions and the alternatively activated (M2, CD163+) macrophages, with anti-inflammatory and tissue repair functions. In the tonsils of pediatric patients with broader expression of viral latency proteins, our group previously revealed a higher presence of CD163+ macrophages, as well as a lower expression of local and peripheral TNF-α [13]. Moreover, we provided evidence that latent and lytic viral antigens influence the expression in macrophages of PD-L1, a costimulatory molecule that induces immune tolerance in T cells [14]. All these findings relate viral antigen expression with regulatory cells and molecules and reveal a peculiar behavior of macrophages in pediatric EBV infection. The higher incidence of EBV-associated lymphomas in children and the key role of macrophages in tumor development and progression [15,16] expose the importance of and need for more studies in the field. In this study, our aim was to characterize peripheral blood monocytes, as macrophages’ precursors, and evaluate their behavior in EBV asymptomatic infection. Understanding the interplay of monocytes, macrophages, and EBV in pediatric patients’ infection might help comprehend the role of innate immunity in EBV infection and, ultimately, clarify viral association with lymphomagenesis in children.

## 2. Materials and Methods

### 2.1. Ethical Statement

All samples were collected after written informed consent, following the national and international ethics standards, and under the supervision of the Ethical Committee of the Ricardo Gutiérrez Children’s Hospital (CEI), in accordance with the Helsinki Declaration of 1975.

### 2.2. Patients and Samples

The cohort included 15 patients (age range 1 to 15 years, median 5), undergoing tonsillectomy due to nonreactive hyperplasia at the Ricardo Gutierrez Children’s Hospital (Buenos Aires, Argentina). Surgery was performed only in the absence of signs or symptoms of an acute illness. At surgery, fresh tissue samples and two concomitant blood samples were collected. The biopsy was formalin-fixed paraffin-embedded (FFPE) at the Pathology Division. From one blood sample, serum was separated to determine the EBV infection status by indirect immunofluorescence assay as previously reported [5]. The cohort included 3 primary infected (PI) patients, 7 healthy carriers (HCs), and 5 patients undergoing reactivation (R).

### 2.3. Immunohistochemistry/Immunofluorescence

Immunohistochemistry (IHC) was performed on tonsil biopsies. Latency viral antigens were assessed using specific primary antibodies for LMP1 (CS1–4 clones, dilution 1/50, Dako, Santa Clara, CA, USA) and EBNA2 (1E6 and R3 clones, dilution 1/5, Abcam, Cambridge, UK), and lytic antigen BMRF1 primary antibodies (G3-E31 clone, dilution 1/200, Abcam) were used as described [5]. Double immunostaining was performed for macrophages’ polarization markers CD68 (clone KP-1, ready to use, Roche Ventana, Oro Valley, AZ, USA) and CD163 (clone MRQ-26, dilution 1/50, Roche Ventana) and PD-L1 (clone 4E54, dilution 1/100, Abcam) as described [14]. Antigen unmasking with sodium citrate buffer (pH 6) was performed in an autoclave for 5 min for LMP1, BMRF1, PD-L1, CD163, and CD68, whereas Tris-EDTA (pH9) was used for EBNA2. Positive cell count was assessed using A1 AxioScope and a Carl Zeiss microscope (Carl Zeiss, Oberkochen, Baden-Württemberg, Germany, and the results were expressed as positive cells per area unit (cells+/mm^2^).

### 2.4. Monocyte Characterization

From peripheral blood samples mononuclear cells were isolated with Lymphoprep (STEM CELL Technologies, Vancouver, BC, Canada). Cells were divided and stained with specific antibodies, namely CD14 PerCP Cy5.5 (HCD14 clone), CD16 PE (3G8 clone), CD64 FITC (10.1 clone), PD-L1 FITC (MIH2 clone), HLA-DR FITC (LN3 clone), CD206 FITC (15–2 clone), CD86 PE (BU63 clone), and CD163 PE (RM3/1 clone) (5 μL per 10^6^ cells, all Biolegend, San Diego, CA, USA), in phosphate-buffered saline-0.5% BSA for 30 min. Cells were then washed, fixed with paraformaldehyde 1%, and analyzed by flow cytometry with a BD FACSCalibur (BD Biosciences, Franklin Lakes, NJ, USA) cytometer. The gating of positive populations for any marker was based on fluorescence minus one (FMO) control. Monocyte subpopulations were identified as classical (C, CD14++CD16−), intermediate (I, CD14++CD16+), and non-classical (NC, CD14+CD16++). CD64, CD86, CD163, CD206, PD-L1, and HLA-DR were evaluated in each subpopulation as indirect functional indicators. Due to technical impediments, CD86 and CD163 were only evaluated in CD14++ as classical and CD14+ as non-classical monocytes. Data were analyzed with FlowJo 10 software (FlowJo, Ashland, OR, USA).

### 2.5. Statistical Analysis

The data were analyzed using GraphPad Prism 9.4 software. Outliers were defined using the Robust test to compare data median absolute deviation (Mad) in Excel. The normality test was performed using the Shapiro–Wilks test. Comparison between groups was assessed by one-way ANOVA or Kruskal–Wallis tests according to normality test results. Multiple comparisons were evaluated with Tukey or Dunn’s correction. Correlations were performed using Spearman or Pearson tests. All tests were two-tailed, and *p* < 0.05 was considered statistically significant.

## 3. Results

### 3.1. Monocyte Subpopulations

**Figure 1:**
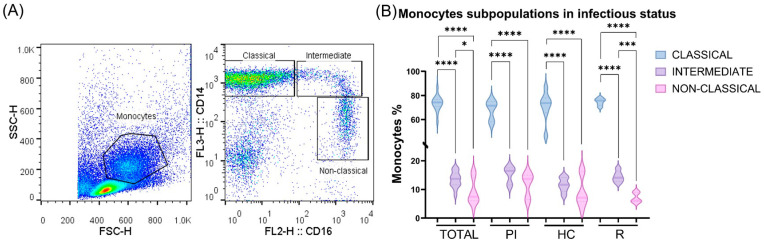
Monocyte subpopulations. Peripheral blood mononuclear cell (PBMC) samples from pediatric patients infected with EBV were stained with CD14 PerCP Cy5.5 and CD16 PE and analyzed by flow cytometry: (**A**) Representative SSC-H vs. FSC-H dot plot with selected monocyte populations and CD14 vs. CD16 dot plot defining monocyte subpopulations are shown. (**B**) Percentage of the different monocyte subpopulations in the entire cohort (total) and in the different stages of viral infection (PI, HC, and R). * *p* < 0.05; *** *p* < 0.001; **** *p* < 0.0001.

To evaluate monocytes in EBV infection, we compared the percentage of each monocyte subpopulation (C, I, and NC) (Figure 1A) between the infectious status (PI, HC, and R), and no significant differences were observed (*p* > 0.05, ANOVA and *t*-test). Then, the mean value of each monocyte subpopulation was compared among PI, HC, and R status. C monocytes were significantly higher than I, and, in turn, both subpopulations were significantly higher than NC when the cohort was analyzed as a whole, and in the PI, HC and R subgroups (*p* < 0.05, ANOVA and *t*-test) (Figure 1B). 

### 3.2. The Expression of Monocytes’ Membrane Markers 

**Figure 2:**
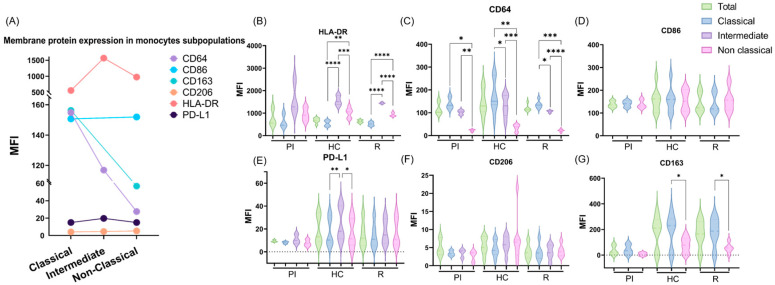
The expression of monocytes’ membrane markers: (**A**) Comparison of membrane protein expression (MFI) between monocyte subpopulations (C, I, and NC) in the entire cohort. Expression of (**B**) HLA-DR, (**C**) CD64, (**D**) CD86, (**E**) PD-L1, (**F**) CD206, and (**G**) CD163 between monocyte subpopulations in each infectious status (PI, HC, and R). * *p* < 0.05; ** *p* < 0.01; *** *p* < 0.001; **** *p* < 0.0001.

The expression of the membrane proteins CD86, CD206, CD163, HLA-DR, CD64, and PD-L1 in each of the three subpopulations was evaluated as indirect indicators of their functions (Figure 2A–G). When the MFI (mean fluorescence intensity) of each marker was compared among C, I, and NC monocytes, CD64 was higher in C, followed by I, and lastly, NC monocytes in the entire cohort, and these results remained the same in the three infectious states (*p* < 0.05; ANOVA) (Figure 2C). PD-L1 expression prevailed in I when the cohort was analyzed as a whole and particularly in HC patients (*p* < 0.05; ANOVA) (Figure 2E). Similarly, HLA-DR was significantly higher in I than in NC monocytes, and these, in turn, were higher than C, in the entire cohort, and in HC and R patients (*p* < 0.05; ANOVA) (Figure 2B). As previously mentioned, CD86 and CD163 were exclusively evaluated in C and NC monocytes. CD163 was significantly higher in C in the entire cohort, as well as in HC and R patients (*p* < 0.05; *t*-test) (Figure 2G). Finally, CD206 and CD86 did not present differences in their expression among monocytes´ subpopulations (C=I=NC) neither in the entire cohort (Figure 2A) nor in infection stages (*p* > 0.05; ANOVA) (Figure 2D,F). On the other hand, the mean expression of each marker (CD86, CD206, CD163, HLA-DR, CD64, and PD-L1) was compared in the entire monocyte population between the different infection stages (PI, P, and R), and no significant differences were demonstrated (*p* > 0.05; ANOVA). In line with this, when these mean values were compared between the infection stages in each monocyte subpopulation in particular (C, I, and NC), no significant differences were found either (*p* > 0.05; ANOVA).

### 3.3. Monocytes and Tonsillar Macrophages

**Figure 3:**
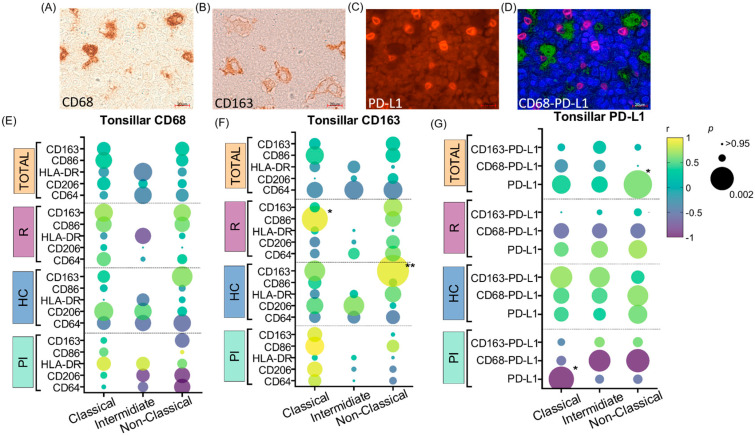
Monocytes and tonsillar macrophages. Representative images (100×) of positive IHC staining in tonsils from pediatric patients infected with EBV of (**A**) CD68, (**B**) CD163, (**C**) PD-L1, (**D**) multiple staining for CD68 and PD-L1 with Hoestch as the nuclear indicator. (**E**) Correlation graph between tonsillar CD68 expression and membrane protein expression (CD163, CD86, HLA-DR, 7 CD206, and CD64) in monocyte subpopulations in the entire cohort and in each infectious status (PI, HC, and R). (**F**) Correlation graph between tonsillar CD163 expression and membrane protein expression (CD163, CD86, HLA-DR, CD206, and CD64) in monocyte subpopulations in the entire cohort and in each infectious status (PI, HC, and R). (**G**) Correlation graph between tonsillar PD-L1 and PD-L1 expressed by macrophages (CD68-PD-L1 and CD163-PD-L1) with PD-L1 expression in monocyte subpopulations in the entire cohort and in each infectious status (PI, HC, and R). The color scale represents Spearman or Pearson correlation coefficient (r); the green-to-yellow colors represent positive r values between 0 and 1, while the blue-to-violet colors represent negative r values between 0 and −1. The size of the dots represents the *p* value, which is larger when the *p* value is smaller. Significant *p* values (*p* < 0.05) are indicated with asterisks. * *p* < 0.05; ** *p* < 0.01.

Given that monocytes can differentiate into macrophages once in tissues, the correlations between monocytes and macrophage polarization markers in the tonsil (CD68 and CD163) were evaluated. CD68+ (Figure 3A) and CD163+ (Figure 3B) cell counts in tissue were correlated with the percentage of C, I, and NC monocytes, as well as with the expression of CD86, CD206, CD163, HLA-DR, and CD64 in the entire monocyte population and in the C, I and NC subpopulations. This analysis was assessed in the entire cohort and in each stage of EBV infection. Significant correlations were not observed between CD68+ cells in tissue and the percentages of monocyte subpopulations or the expression of the functional markers evaluated in each of them (*p* > 0.05; Spearman and Pearson) (Figure 3E). Regarding CD163+ cells in tissue, positive correlations were found, on the one hand, with the expression of CD86 in total monocytes in R patients (*p* = 0.0365; r = 0.9016; Pearson) and particularly in C monocyte subtype (*p* = 0.0365; r = 0.9016; Pearson), and, on the other, with the expression of CD163 in NC monocytes in HC patients (*p* = 0.0042; r = 0.9124; Pearson) (Figure 3F). In addition, since our group previously described a peculiar upregulation of PD-L1 in M2 macrophages in the presence of LMP-1 viral protein [14], we compared PD-L1 expression in monocytes with total PD-L1 expression in tonsils (Figure 3C) and with PD-L1 expressed in macrophages (CD68-PD-L1+ and CD163-PD-L1+) (Figure 3D). A significant negative correlation was demonstrated between all PD-L1+ cells in tissue and the expression of PD-L1 in C monocytes, exclusively in PI patients (*p* = 0.0372; r = −0.998; Pearson). In contrast, a positive correlation between PD-L1+ cells in tissue and PD-L1 expression in NC monocytes in the entire cohort of patients was confirmed (*p* = 0.0162; r = 0.6179; Spearman) (Figure 3G). When the expression of PD-L1 in monocytes was compared with the expression of PD-L1 specifically in macrophages, no significant differences were demonstrated.

### 3.4. Monocytes and Viral Antigen Expression

**Figure 4:**
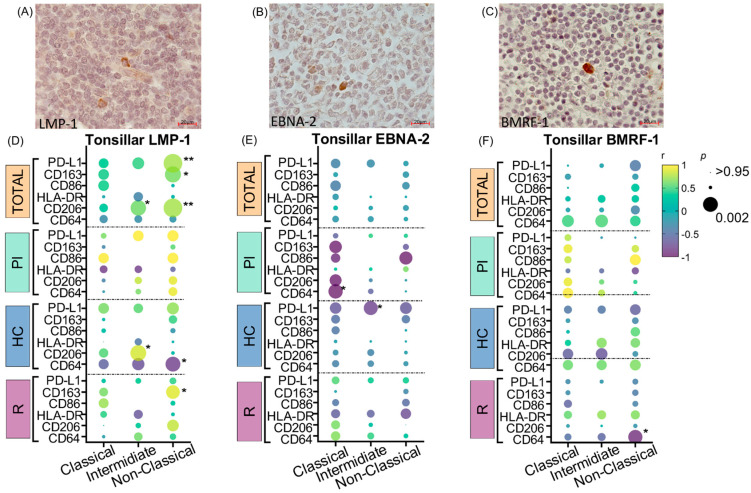
Monocytes and viral antigen expression. Representative images (100×) of positive IHC staining in tonsils from pediatric patients infected with EBV of (**A**) LMP-1, (**B**) EBNA-2, and (**C**) BMRF-1. (**D**) Correlation graph between tonsillar LMP-1 expression and membrane protein expression (CD163, CD86, HLA-DR, CD206, and CD64) in monocyte subpopulations in the entire cohort and in each infectious status (PI, HC, and R). (**E**) Correlation graph between tonsillar EBNA-2 expression and membrane protein expression (CD163, CD86, HLA-DR, CD206, and CD64) in monocyte subpopulations in the entire cohort and in each infectious status (PI, HC, and R). (**F**) Correlation graph between tonsillar BMRF-1 expression and membrane protein expression (CD163, CD86, HLA-DR, CD206, and CD64) in monocyte subpopulations in the entire cohort and in each infectious status (PI, HC, and R). The color scale represents Spearman or Pearson correlation coefficient (r); the green-to-yellow colors represent positive r values between 0 and 1, while the blue-to-violet colors represent negative r values between 0 and −1. The size of the dots represents the *p* value, which is larger when the *p* value is smaller. Significant *p* values (*p* < 0.05) are indicated with asterisks. * *p* < 0.05; ** *p* < 0.01.

In a previous work, we described a particular association between EBV viral proteins and regulatory macrophages [13,14]. To assess whether the virus exerts an influence on the different peripheral monocyte populations and/or their membrane markers, lytic and viral latency proteins’ positive cell counts in tonsils were correlated with the percentages of C, I, and NC monocytes and with the expression of CD86, CD206, CD163, HLA-DR, CD64, and PD-L1, both in the entire monocyte population and in the C, I, and NC subpopulations. This analysis was performed in the cohort as a whole and in groups with different infectious status. Tonsillar LMP-1+ (Figure 4A) cells exhibited significant positive correlations with PD-L1, CD206, and CD163 expression in NC monocytes (*p* = 0.0022; r = 0.7417; Spearman; *p* = 0.0021; r = 0.7484; Pearson; *p* = 0.0105; r = 0.6381; Pearson, respectively) in the whole cohort and with CD163 particularly in R patients (*p* = 0.0367; r = 0.9013; Pearson). Moreover, in I monocytes as well, LMP-1 correlated with CD206 in all the patients and specifically in HC patients (*p* = 0.0146; r = 0.6152; Pearson). Furthermore, LMP-1 negatively correlated with CD64 expression on NC monocytes in HC patients (*p* = 0.0205; r = −0.8311; Pearson) (Figure 4D). Regarding tonsillar EBNA-2+ cells (Figure 4B), exclusively in PI, negative correlations with the expression of CD64 in the C monocytes (*p* = 0.0295; r = −0.9979; Pearson) and CD163 in the total monocytes (*p* = 0.0410; r = −0.9979; Pearson) were observed. In addition, EBNA-2 inversely correlated with PD-L1 in I (*p* = 0.0351; r = −0.7885; Pearson) in HC patients and with HLA-DR of total monocytes in R patients (*p* = 0.0175; r = −0.9399; Pearson) (Figure 4E). Finally, BMRF-1 (Figure 4C) presented a negative correlation with the expression of CD64 in NC monocytes in R patients (*p* = 0.0271; r = −0.9196; Pearson) (Figure 4F).

## 4. Discussion

Since discovered in Burkitt lymphoma, the correlation between EBV infection and tumor development has widely been described, based on the oncogenic potential of many viral proteins [17]. Since tumor development can be related to an imbalance between EBV latent and lytic protein activity and the immune system, immune response characterization against EBV infection has been the focus of many studies [18,19]. However, due to the difficulty in detecting asymptomatic infections, most studies characterize the immune response in the context of infectious mononucleosis in adult patients. The few studies that characterize silent infections describe a key role of the innate system, in particular NK cells [20]. In Argentina, EBV primary infection occurs at young ages (3–4 years old) and is mostly asymptomatic, whereas EBV-associated lymphomas are significantly increased in children younger than 10 years [6]. As far as we know, this is the first study that characterized monocytes in pediatric EBV infection and its association with tonsil macrophages.

When monocyte subpopulations were characterized, we observed that monocyte subpopulations in our cohort of patients with EBV were similar to that described in healthy patients, both pediatric and adults [21,22], where C monocytes prevailed, followed by I, and lastly NC, suggesting that EBV infection would not alter monocyte subpopulations. To deepen the analysis and understand the role of monocytes in EBV infection in more detail, the expression of different membrane proteins in each subpopulation as indirect functionality markers were analyzed. CD86 and CD206 showed homogeneous expression among C, I, and NC monocytes, across all EBV infectious statuses, opposite to what was previously described in inflammatory diseases [23,24,25]. Since higher expressions of CD86 and CD206 were previously demonstrated on NC monocytes, this might indicate that EBV could either upregulate CD86 and CD206 expression in CD16-monocytes or downregulate both of them in CD16+ monocytes [23,24,25]. When CD163 expression was evaluated, a broader expression in C compared to NC monocytes was demonstrated, similar to what was observed in chronic B hepatitis infection [26]. Lastly, PD-L1 expression was higher in I monocytes when patients were analyzed as a whole, and in particular in HC patients. In line with this, it has been demonstrated that PD-L1 expression prevails in I monocytes, not only in healthy adults but also during HIV infection and tuberculosis (TB), although the higher expression of PD-L1 was demonstrated to be in the context of HIV/TB coinfection [27]. Hence, PD-L1 expression in monocytes would be influenced by EBV mainly in the context of persistent infection in HCs.

To further explore the interplay between EBV and monocytes, the correlation between latent and lytic viral protein expression in the tonsils with monocytes’ membrane protein was evaluated, revealing a positive correlation between tonsillar LMP-1 and PD-L1, CD206, and CD163 expression in NC monocytes. These findings might suggest that the main viral oncoprotein modulates the expression of immunoregulatory markers in monocytes and indicates the presence of a permissive environment in a less inflammatory microenvironment. In fact, a local upregulation of PD-L1 in the presence of LMP-1 was described in HL [28] and nasopharyngeal carcinoma [29]. Moreover, LMP-1 negatively correlated with the expression of CD64 in NC monocytes in HC patients, favoring a less inflammatory environment not only by upregulating regulatory molecules but also by downregulating proinflammatory ones. Contradictorily, tonsillar EBNA-2 cell count negatively correlated with PD-L1 expression in I monocytes in HC patients, whereas lytic infection seems to have a slight effect, reinforcing the concept of LMP-1 as the main viral protein involved in tumorigenesis, even in the context of latency III protein expression, as previously suggested [30]. Remarkably, we found that the I and NC subpopulations, which have been associated with antiviral functions, exhibited associations with viral proteins [10].

EBV primary infection and reactivation occurs in the tonsil. Our group has been studying the macrophage response against EBV in this essential structure for the viral life cycle. Since peripheral monocytes are recruited and differentiate into macrophages in, among others, tonsillar tissue, we characterized the possible connection between them. A positive correlation between tissue and monocyte CD163 markers was demonstrated, even though this correlation was exclusively for NC monocytes in HC patients. These results might indicate that the recruitment of NC CD163+ monocytes to the tonsil during persistent infection is likely to differentiate into CD163+ macrophages. Considering the results described above, the NC subpopulation seems to have a relevant role in EBV infection, particularly in HC patients, as for other viral infections. Moreover, when tonsillar PD-L1 expression was correlated with PD-L1 expression by monocytes, a positive correlation was demonstrated only for the NC subpopulation. It could be concluded that the contribution of PD-L1 to the tissue and its immunoregulatory effect by monocytes would be primarily through the migration of NC monocytes, the subpopulation of monocytes that respond primarily to viral infections [10]. On the other hand, unexpectedly, no correlation between double CD68+PD-L1+ or CD163+PD-L1+ cells and PD-L1 expression in monocytes could be demonstrated. In a previous study, our group demonstrated that, although PD-L1 expression was upregulated in CD163+ macrophages in pediatric patients with broader LMP-1 viral protein expression, macrophages are not the PD-L1+ predominant cells [14]. Taken together, these findings, in addition to the fact that monocytes have the ability to also differentiate into dendritic cells [31], it might be speculated that dendritic cells could be the cells with the greatest contribution of PD-L1 expression in the tonsils of pediatric patients with EBV.

## 5. Conclusions

This work aims to contribute to the knowledge about the role of monocytes/macrophages in the context of EBV asymptomatic infection in children, and ultimately, in the pathogenesis of pediatric EBV-associated lymphomas. Even though the patient series is small to draw definitive conclusions, and additional studies with larger cohorts in diverse populations are needed, our results suggest a predominant role of I and NC monocyte subpopulation response against EBV infection, with even more relevant participation observed in the HC stage, as established in other persistent viral infections. Furthermore, the viral oncoprotein LMP-1 could be involved in the expression of regulatory proteins in I and NC monocytes.

## Data Availability

The datasets generated during and/or analyzed during the current study are available from the corresponding author upon reasonable request.
